# ALDH2 regulates oxaliplatin sensitivity via PDE4B in p53-mutant colorectal cancer

**DOI:** 10.7150/ijms.133461

**Published:** 2026-07-27

**Authors:** Po-Li Wei, Chien-Yu Huang, Cheng-Chin Lee, Uyanga Batzorig, Kuei-Yen Tsai, Yu-Jia Chang

**Affiliations:** 1 Department of Surgery, School of Medicine, College of Medicine, Taipei Medical University, Taipei 11031, Taiwan.; 2 Division of Colorectal Surgery, Department of Surgery, Taipei Medical University Hospital, Taipei Medical University, Taipei 11031, Taiwan.; 3 Cancer Research Center and Translational Laboratory, Department of Medical Research, Taipei Medical University Hospital, Taipei Medical University, Taipei 11031, Taiwan.; 4 Graduate Institute of Cancer Biology and Drug Discovery, Taipei Medical University, Taipei 11031, Taiwan.; 5 School of Medicine, National Tsing Hua University, Hsinchu 300044, Taiwan.; 6 Institute of Molecular and Cellular Biology, National Tsing Hua University, Hsinchu 300044, Taiwan.; 7 Department of Pathology, Wan Fang Hospital, Taipei Medical University, Taipei 11031, Taiwan.; 8 Graduate Institute of Clinical Medicine, College of Medicine, Taipei Medical University, Taipei 11031, Taiwan.; 9 Department of Dermatology, University of California, San Diego, La Jolla, CA 92093, USA.; 10 Division of General Surgery, Department of Surgery, Shuang Ho Hospital, Taipei Medical University, New Taipei City 235041, Taiwan.; 11 Cell Physiology and Molecular Image Research Center, Wan Fang Hospital, Taipei Medical University, Taipei 11031, Taiwan.

**Keywords:** Chemoresistance, p53-mutant colorectal cancer, Aldehyde dehydrogenase 2, p53-mutant, p53-wild-type

## Abstract

**Background:**

Chemoresistance remains a major limitation in the treatment of colorectal cancer (CRC), particularly in tumors harboring TP53 mutations. Although aldehyde dehydrogenase 2 (ALDH2) has been implicated in cancer biology, its role in regulating platinum sensitivity and the underlying molecular context remain incompletely understood.

**Methods:**

Public transcriptomic and proteomic datasets (TCGA, GEO, CPTAC) were analyzed to evaluate ALDH2 expression patterns, prognostic relevance, and associations with chemotherapy response in CRC. Functional studies were performed in p53-mutant and p53-wild-type CRC cell lines using pharmacological inhibition and genetic silencing of ALDH2, complemented by p53 knockout and reconstitution approaches. Downstream signaling was interrogated by gene set enrichment analysis, quantitative PCR, and pharmacological inhibition of phosphodiesterase 4B (PDE4B). Oxaliplatin sensitivity was assessed using cell viability and apoptosis assays.

**Results:**

ALDH2 expression was significantly reduced in CRC tissues and was associated with unfavorable clinical outcomes and diminished oxaliplatin responsiveness. Functional experiments revealed that inhibition or knockdown of ALDH2 selectively induced oxaliplatin resistance in p53-mutant CRC cells, while p53-wild-type cells were largely unaffected. Mechanistically, loss of ALDH2 led to upregulation of PDE4B in a p53 status-dependent manner. Elevated PDE4B expression correlated with chemoresistance and poor recurrence-free survival in patients with p53-mutant CRC. Importantly, pharmacological inhibition of PDE4B using the clinically approved drug roflumilast effectively restored oxaliplatin-induced apoptosis and reversed chemoresistance in ALDH2-deficient, p53-mutant CRC cells.

**Conclusions:**

This study identifies a previously unrecognized, p53 status-dependent ALDH2-PDE4B regulatory axis that governs oxaliplatin sensitivity in colorectal cancer. Our findings highlight ALDH2 as a potential predictive biomarker for oxaliplatin response in p53-mutant CRC and suggest that targeting PDE4B may represent a promising strategy to overcome chemoresistance in this molecular context.

## Introduction

Colorectal cancer (CRC) remains one of the leading causes of cancer-related mortality worldwide 1. Despite substantial advances in surgical techniques and systemic therapies, platinum-based chemotherapy, particularly oxaliplatin, continues to constitute a cornerstone of treatment for patients with advanced disease 2, 3. However, intrinsic and acquired resistance to oxaliplatin markedly limits its clinical efficacy and contributes to disease recurrence and poor outcomes 4. Identifying molecular determinants that govern oxaliplatin sensitivity is therefore critical for improving therapeutic stratification and treatment outcomes in CRC.

Among genetic alterations in CRC, mutations in TP53 are especially prevalent and clinically consequential 5. Beyond its canonical role in DNA damage responses and apoptosis, p53 has emerged as a key regulator of cellular metabolism and stress adaptation 6, 7. Accumulating evidence indicates that p53 mutation status profoundly influences therapeutic responses, including resistance to fluoropyrimidines, platinum agents, and targeted therapies 8. Nevertheless, the upstream metabolic regulators and downstream signaling effectors through which p53 status shapes chemoresistance remain incompletely defined.

Aldehyde dehydrogenase 2 (ALDH2) is a mitochondrial enzyme responsible for detoxifying reactive aldehydes generated during oxidative and metabolic stress 9. By limiting aldehyde-induced DNA damage and maintaining redox homeostasis, ALDH2 has been implicated in genome stability and tumor suppression 10, 11. Emerging studies suggest that ALDH2 expression is frequently reduced in colorectal cancer and may influence tumor progression and therapeutic responses 12. Notably, our previous work demonstrated that pharmacological inhibition of ALDH2 selectively reduced oxaliplatin sensitivity in p53-mutant CRC cells 13, whereas p53-wild-type cells were largely unaffected. These observations raised the possibility that ALDH2 functions as a context-dependent regulator of chemotherapy response in CRC.

Despite these insights, the molecular mechanisms linking ALDH2 activity to oxaliplatin sensitivity, and the reasons for its strict dependence on p53 status, have not been elucidated. In particular, whether ALDH2 engages downstream signaling pathways that are differentially wired according to p53 context remains unknown. Addressing this knowledge gap is essential for understanding how metabolic regulation intersects with genetic alterations to drive chemoresistance.

In the present study, we combine integrative analyses of public clinical datasets with functional genetic and pharmacological experiments to define the role of ALDH2 in regulating oxaliplatin sensitivity in colorectal cancer. We identify phosphodiesterase 4B (PDE4B) as a previously unrecognized downstream effector of ALDH2 and demonstrate that an ALDH2-PDE4B regulatory axis operates selectively in p53-mutant CRC cells. Furthermore, we show that pharmacological inhibition of PDE4B using the clinically approved drug roflumilast restores oxaliplatin-induced apoptosis and reverses chemoresistance in this molecular context. Together, these findings establish a novel p53 status-dependent metabolic-signaling axis that governs oxaliplatin response and highlight actionable vulnerabilities in p53-mutant colorectal cancer.

## Materials and Methods

### Survival analysis

The study used the online tool Kaplan-Meier plotter to evaluate data from CRC patients in the Gene Expression Omnibus (GEO) database, analyze the prognostic value of ALDH2 gene expression in patients, and estimate the correlation between gene expression and survival in colorectal cancer (https://kmplot.com/analysis/). Patient samples were divided into two groups based on median mRNA expression, and log-rank p-values, hazard ratios (HRs), and 95% confidence intervals (95% CIs) were calculated. Furthermore, COAD (colon adenocarcinoma) RNA sequence data (FPKM) and clinical information from the TCGA database (https://portal.gdc.cancer.gov) were processed using the STAR workflow. Kaplan-Meier analysis was performed using the Survival 3.3.1 package, and results were visualized using survminer and ggplot2. Statistical significance was defined as a p-value less than 0.05.

### Gene and protein expression levels of ALDH2 in colon cancer database

UALCAN, developed by the University of Alabama at Birmingham, serves as a web-based platform that facilitates the investigation of genomic expression patterns, proteomic profiles in malignancies, and clinical outcome information derived from TCGA and the Clinical Proteomic Tumor Analysis Consortium (CPTAC) repositories 14. In addition, we used TNMplot, a web-based bioinformatics tool designed for analyzing gene expression data across different cancer types, to compare RNA expression levels between tumor tissues and normal tissues. These resources, available at http://ualcan.path.uab.edu, and https://tnmplot.com/analysis/, were utilized to examine the association between ALDH2 expression and colorectal cancer.

### Gene set enrichment functional analysis

The study used the DESeq2 1.36.0 suite to analyze differential gene expression between ALDH2 high and low expression groups in the TCGA COAD dataset 15. Differentially expressed genes (DEGs) were identified using an adjusted logFC ≥ 1 and a p-value ≤ 0.05. Gene Set Enrichment Analysis (GSEA) was performed using the clusterProfiler 4.4.4 suite 16, and the results were visualized using the ggplot2 3.3.6 suite.

### Analysis of chemotherapeutic response

The study used the ROC plotter (https://www.rocplot.org/) to assess the sensitivity and specificity of ALDH2 as a classifier for analyzing chemotherapy response in CRC patients. Boxplots visually displayed ALDH2 expression levels in responders and non-responders. Additionally, the area under the curve (AUC) and corresponding p-values were calculated, and the Mann-Whitney U test was used to assess the statistical significance of the observed differences.

### Cell cultivation and reagents

The colorectal cancer cell lines DLD-1 (RRID: CVCL_0248), HT-29 (RRID: CVCL_0320), and HCT 116 (RRID: CVCL_0291) were sourced from the American Type Culture Collection. These cells were cultured in RPMI medium containing 10% fetal bovine serum (FBS) and 1% penicillin-streptomycin under standard conditions (37 °C, 5% CO_2_, humidified atmosphere). They were also tested negative for mycoplasma contamination. Passaging was performed biweekly upon reaching 80% confluence. The chemotherapeutic drug oxaliplatin (Oxalip, TTY Biopharm) was used in this study. The compound daidzein (catalog No. S1849) was procured from Selleck Chemicals LLC. Roflumilast was obtained from Sigma-Aldrich (Ref. SML1099). DMSO served as the solvent for daidzein and roflumilast.

### Examination of cell viability

The CCK-8 assay (Dojindo Laboratories, Kumamoto, Japan) was employed to assess cellular viability. Colorectal cancer cells were plated at a density of 15×10³ cells per well in 96-well culture plates. Following a 24-hour exposure to varying doses of oxaliplatin, either alone or in combination with daidzein (0, 50, and 100 μM) or roflumilast (0 and 100 μM), 100 μL of CCK-8 reagent was introduced to each well. After a 2-hour incubation period at 37 °C, absorbance readings were obtained at 450 nm wavelength using a microplate spectrophotometer (Bio-Rad Laboratories, Hercules, CA, USA).

### Knockout of p53 and restoration in cell lines

HCT 116 p53 (-/-) cells, originally derived from Johns Hopkins University (Baltimore, MD, USA), were used in this study. To reconstitute p53 expression, wild-type p53 or mutant p53 (S241F) complementary DNA was cloned into the pTwist cDNA 3.1 overexpression vector (Twist Bioscience, South San Francisco, CA, USA). The constructed plasmids were transfected into HCT 116 p53 (-/-) cells using electroporation according to the manufacturer's protocol (Neon Transfection System Model MPK5000, Life Technologies). Transfected cells were cultured in RPMI-1640 medium supplemented with 10% FBS and 1% penicillin-streptomycin at 37 °C in a humidified incubator with 5% CO₂ and used for subsequent experiments.

### Annexin V-FITC/Propidium Iodide (PI) assay

Apoptotic cell detection was conducted using a commercially available annexin V assay kit (Oncogene Research Products, Calbiochem, MA, USA). A total of 5×10⁵ cells were suspended in 500 μL of culture medium and mixed with 10 μL of the media-specific binding reagent along with 1.25 μL of annexin V (200 μg/ml) conjugated to FITC. The mixture was then kept in darkness at room temperature for 10 minutes. Subsequently, the sample underwent centrifugation at 1000 g for 5 minutes under ambient temperature conditions, after which the cell pellet was resuspended in 500 μL of 1× binding buffer containing 10 μL of propidium iodide. This cell suspension was immediately transferred to ice, protected from light exposure, and subjected to flow cytometric analysis without delay. Using the FacScan cytometer operating in low-flow mode, 20,000 cells were captured for analysis. The FL1 and FL2 filters were utilized to detect green and red fluorescence signals, respectively, with data presentation in logarithmic scale. To ensure accurate annexin V/PI measurement, electronic compensation was applied by subtracting 25% of the FL-2 signal from FL-1. Data processing and interpretation were performed using LSYSTEM II software.

### Quantitative reverse-transcription polymerase chain reaction

Total RNA isolation from cultured cells was performed using TRIZOL reagent (Invitrogen Life Technologies, Carlsbad, CA, USA) following the manufacturer's protocol. For cDNA synthesis, 8 μg of isolated RNA underwent reverse transcription utilizing the cDNA Synthesis Kit (Invitrogen Life Technologies) with 1 μL of 12-20 nt oligo(dT) primers, according to the provided guidelines. Real-time PCR amplification was carried out using the ABI 7500 FAST Real-Time PCR System in conjunction with the SYBR Green Master Mix (Applied Biosystems). Each PCR reaction was assembled in a 30 μL total volume containing 1.5 μL cDNA template, 12 μL nuclease-free water, 12 μL SYBR Green Master Mix, and 1.5 μL each of forward and reverse primers. Following thorough mixing, 10 μL aliquots were distributed into individual wells of a 96-well plate. The thermal protocol consisted of an initial 95 °C hold for 10 minutes, followed by 45 amplification cycles (95 °C denaturation for 15 seconds and 60 °C annealing/extension for 1 minute). To ensure reproducibility and account for variations in RNA input quality and processing efficiency, each sample underwent triplicate testing, and the complete experimental series was repeated three times on different occasions.

### Statistical analysis

Each experimental procedure was performed in triplicate or more. Results from RT-PCR analyses and cell proliferation studies are expressed as mean ± standard error of the mean (SEM). Data were derived from at least three independent biological experiments. Statistical comparisons between two groups were evaluated using Student's t-test. In the figures, asterisks indicate the level of statistical significance as follows: *p < 0.05, **p < 0.01, and ***p < 0.001 (specific p-values are provided in the figure legends for detailed reference).

## Results

### ALDH2 expression is reduced in colorectal cancer and correlates with poor clinical outcomes

**Figure 1:**
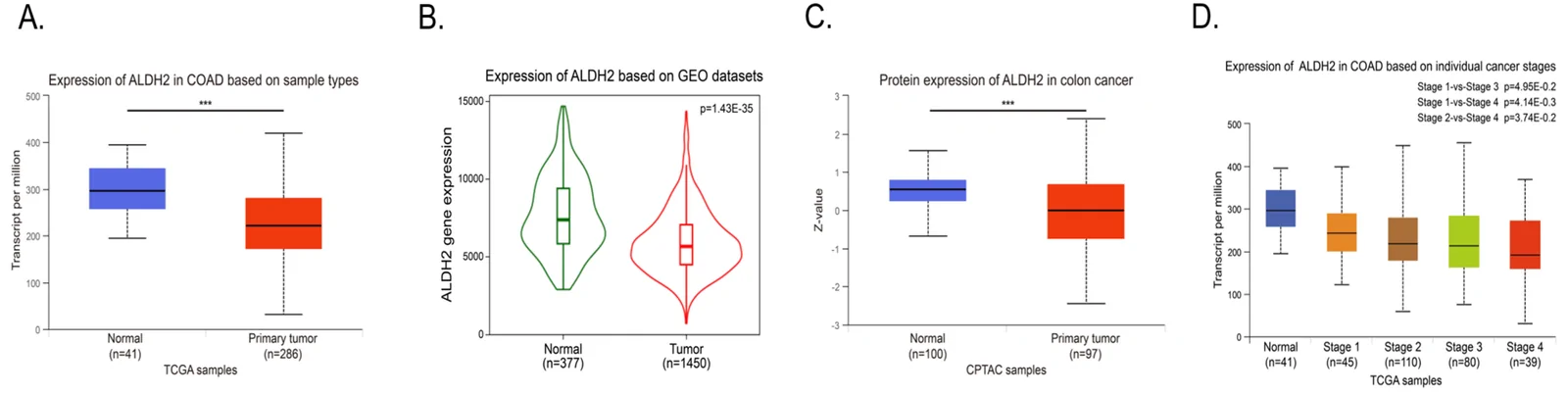
** ALDH2 displayed lower expression in colon cancer tissues compared to normal tissues.** Analysis of ALDH2 mRNA expression in colon adenocarcinoma (COAD) and normal tissues from (A) UALCAN and (B) TNMplot resources. (C) Protein levels of ALDH2 in colon cancer as seen via UALCAN, sourced from the Clinical Proteomic Tumor Analysis Consortium database. The Z-value represents the standard deviation from the median across samples. (D) Expression of ALDH2 in COAD based on individual cancer stages. *** p < 0.001.

Analysis of transcriptomic and proteomic datasets demonstrated that ALDH2 expression was significantly decreased in colorectal cancer tissues compared with adjacent normal mucosa. Consistent reductions in ALDH2 mRNA were observed across TCGA and GEO cohorts, and decreased ALDH2 protein abundance was confirmed using CPTAC data** (Figure 1A-C)**. Moreover, ALDH2 expression progressively declined with advancing tumor stage, supporting a tumor-suppressive role for ALDH2 in colorectal cancer **(Figure 1D)**.

**Figure 2:**
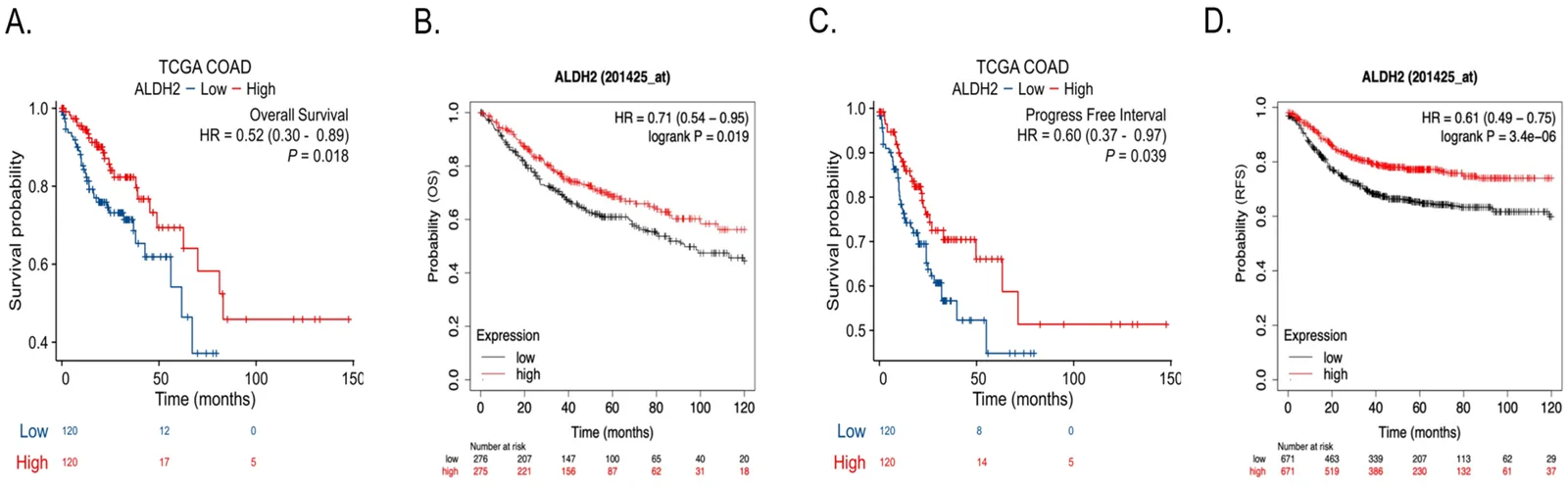
** CRC patients with low ALDH2 expression had a poorer prognosis.** The overall survival rates stratified by ALDH2 expression in patients based on TCGA COAD data (A) and Kaplan-Meier plotter (B). The progression-free intervals were analyzed, showing consistent trends (C & D).

Survival analyses further revealed that patients with low ALDH2 expression exhibited significantly poorer overall survival and progression-free survival compared with those expressing higher ALDH2 levels** (Figure 2A-D)**. These findings establish a strong clinical association between ALDH2 downregulation and adverse oncological outcomes in colorectal cancer.

### Low ALDH2 expression predicts reduced oxaliplatin responsiveness in colorectal cancer

**Figure 3:**
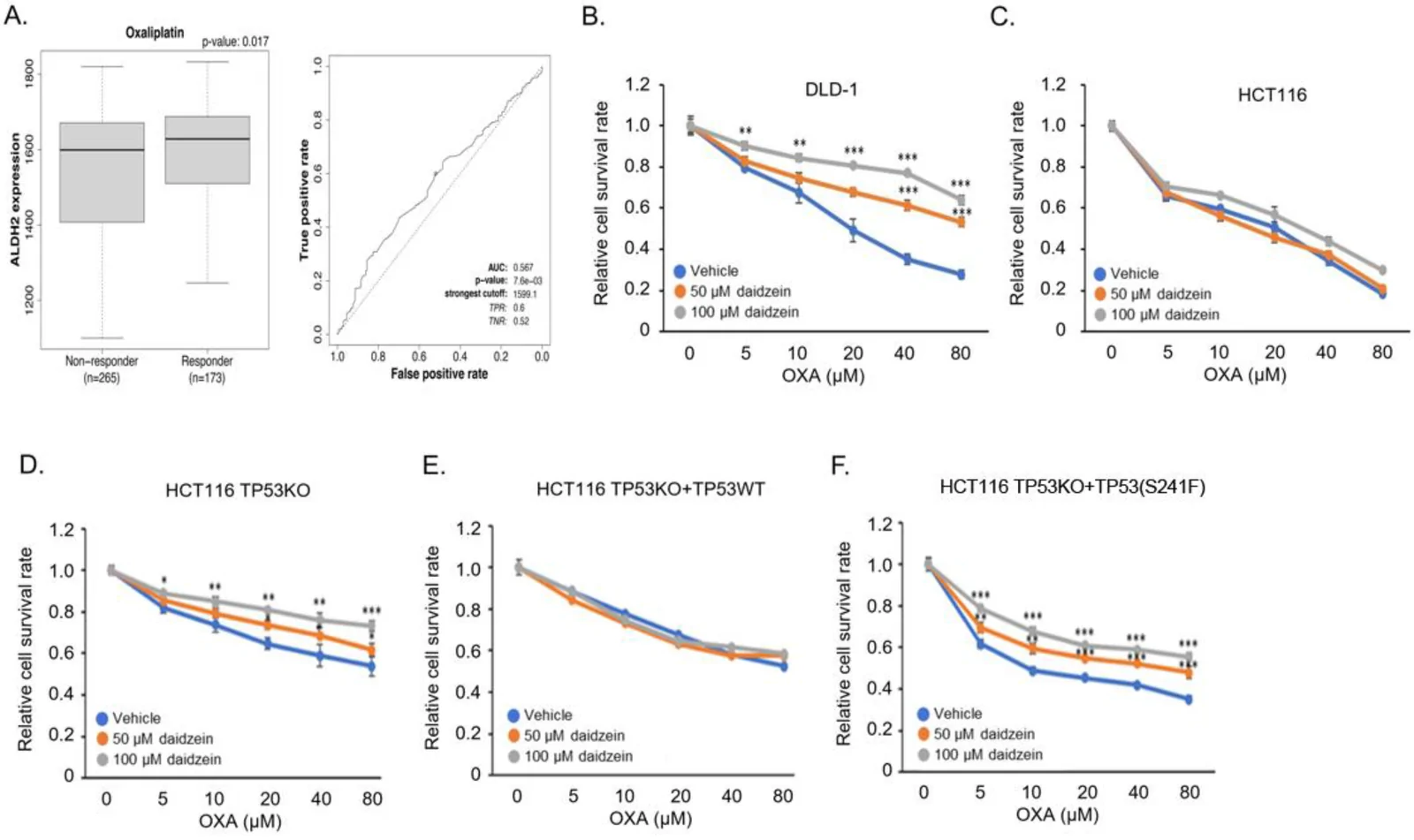
** ALDH2 inhibition induced oxaliplatin resistance in a p53-dependent manner.** (A) Box plots illustrated the expression of ALDH2 in CRC patients stratified by chemotherapy responses according to the Response Evaluation Criteria in Solid Tumors (RECIST) criteria. Receiver operating characteristic (ROC) analysis was conducted to assess the predictive value of ALDH2 expression for treatment response. (B) Co-administering daidzein improved p53 mutant DLD-1 cell viability in a dose-dependent pattern. (C) HCT 116 cells exhibited no significant changes in viability when treated with ALDH2 inhibitor. (D) Knocking out p53 augmented proliferation of HCT 116 cells by daidzein co-treatment in a concentration-dependent fashion. (E) Re-expression of p53 in HCT 116 p53 -/- abolished the ALDH2 inhibitor's effect on oxaliplatin sensitivity. (F) Transfection with mutant p53 did not produce a reversal effect. All experiments were conducted in triplicate. *p < 0.05, **p<0.01, ***p<0.001.

To evaluate whether ALDH2 expression is associated with chemotherapy response, ROC plotter analysis was performed in colorectal cancer patients receiving standard cytotoxic regimens **(Figure 3A)**. ALDH2 expression was significantly lower in oxaliplatin non-responders compared with responders, whereas no significant difference was observed in patients treated with 5-fluorouracil **(Figure S1).** Receiver operating characteristic analysis indicated that ALDH2 expression possessed modest but significant discriminatory power for oxaliplatin response, suggesting a selective association between ALDH2 status and platinum sensitivity.

### Inhibition of ALDH2 selectively induces oxaliplatin resistance in p53-mutant colorectal cancer cells

To functionally validate the role of ALDH2 in oxaliplatin sensitivity, colorectal cancer cell lines with distinct p53 status were treated with oxaliplatin in the presence or absence of the ALDH2 inhibitor daidzein. Oxaliplatin treatment alone reduced cell viability in a dose-dependent manner in both p53-mutant DLD-1 cells and p53-wild-type HCT 116 cells. Notably, co-treatment with daidzein significantly increased survival of DLD-1 cells in a concentration-dependent fashion **(Figure 3B)**, whereas HCT 116 cells remained largely unaffected **(Figure 3C)**.

To directly assess the contribution of p53 status, p53 was genetically ablated in HCT 116 cells. In TP53-deficient HCT 116 cells, ALDH2 inhibition recapitulated the resistance phenotype observed in DLD-1 cells **(Figure 3D)**, indicating that loss of functional p53 is sufficient to unmask ALDH2-dependent oxaliplatin resistance. Conversely, reconstitution of wild-type p53, but not mutant p53, restored oxaliplatin sensitivity and abolished the protective effect conferred by ALDH2 inhibition **(Figure 3E & F)**. Collectively, these findings demonstrate that ALDH2 regulates oxaliplatin sensitivity in a p53 status-dependent manner.

### Genetic silencing of ALDH2 suppresses oxaliplatin-induced apoptosis in p53-mutant CRC cells

**Figure 4:**
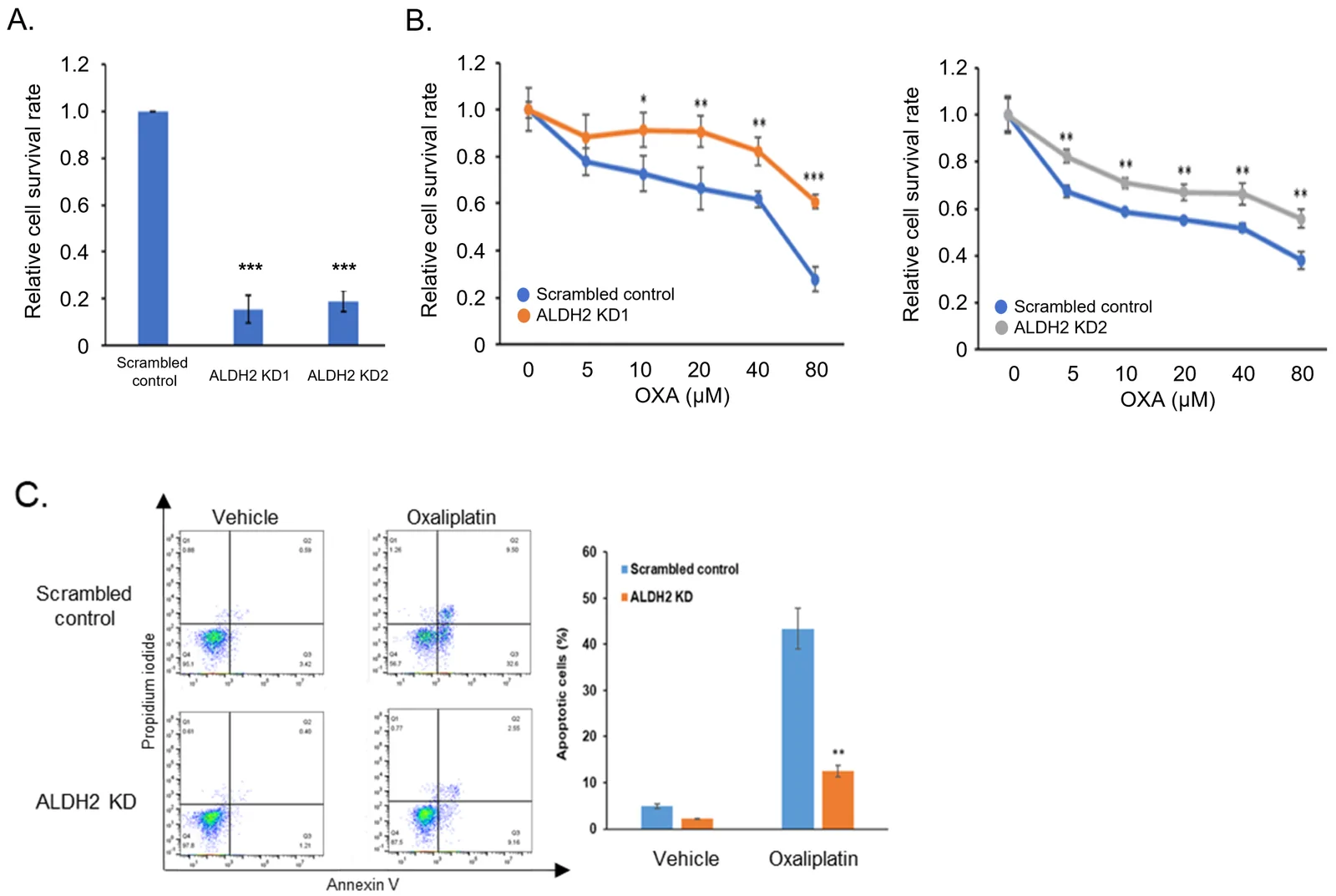
** Silencing ALDH2 reduced oxaliplatin sensitivity and oxaliplatin-mediated apoptosis.** (A) Confirmation of ALDH2 knockdown using RT-qPCR. (B) ALDH2-knockdown increased relative cell survival in DLD-1 cells compared to control under oxaliplatin treatment. (C) ALDH2 silencing suppressed oxaliplatin-induced apoptosis in CRC cells following 24-hour oxaliplatin treatment. All experiments were conducted in triplicate. *p < 0.05, **p<0.01, ***p<0.001.

To exclude potential off-target effects of pharmacological inhibition, ALDH2 expression was silenced using shRNA. Efficient knockdown of ALDH2 was confirmed at the transcript level **(Figure 4A)**. Consistent with inhibitor-based findings, ALDH2 depletion significantly increased cell survival in p53-mutant DLD-1 cells exposed to oxaliplatin **(Figure 4B)**. Flow cytometric analysis further revealed that ALDH2 knockdown markedly attenuated oxaliplatin (80 μM)-induced apoptosis, while basal apoptotic rates were unaffected in the absence of drug treatment **(Figure 4C)**. Importantly, although daidzein is a known phytoestrogen with potential off-target activity, the concordance between daidzein co-treatment results **(Figure 3)** and siRNA-mediated ALDH2 knockdown results **(Figure 4)** strongly supports the conclusion that the observed drug resistance is attributable to ALDH2 inhibition rather than non-specific daidzein effects. These results indicate that ALDH2 loss confers oxaliplatin resistance primarily through suppression of apoptosis in p53-mutant colorectal cancer cells.

### Identification of PDE4B as a downstream effector associated with ALDH2 loss and chemoresistance

**Figure 5:**
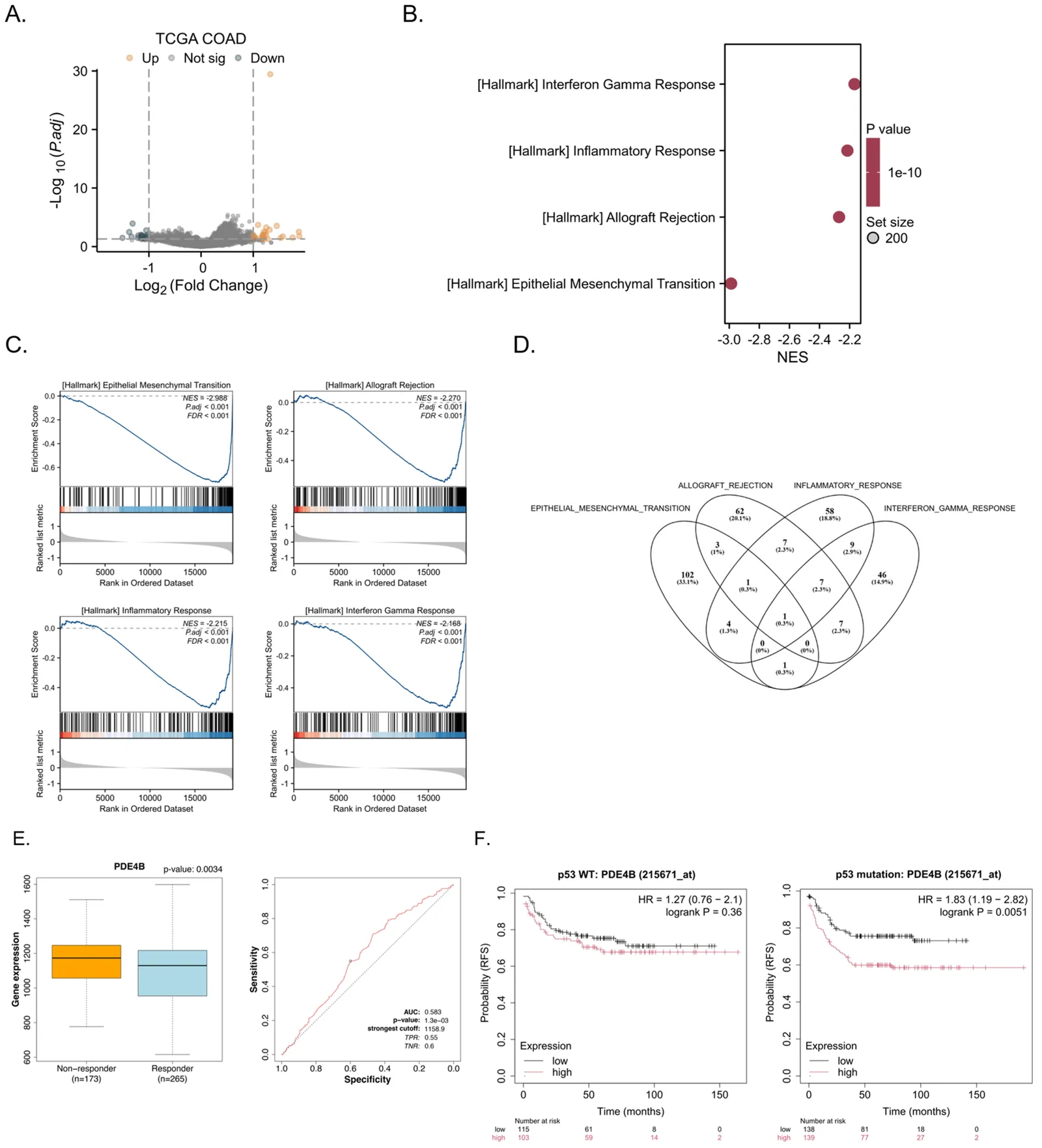
** GSEA results based on ALDH2 expression levels and identification of downstream regulator PDE4B.** (A) Volcano plot of differential expression genes between high (top 50%) - and low (bottom 50%) ALDH2 expression from p53-mutant colorectal cancer patients. (B) The four most significantly enriched pathways determined by GSEA with criteria as padj < 0.05 and NES < -2.1. (C) GSEA enrichment plots of the HALLMARK gene sets. (D) The intersection of any two of the four pathways that were significantly enriched with ALDH2 high and low expression revealed a total of 40 genes. (E) High PDE4B expression was associated with reduced oxaliplatin sensitivity. (F) Expression of PDE4B was correlated with worse recurrence-free survival in patients with p53 mutation, whereas p53 wild-type patients exhibited no significant survival changes regarding PDE4B expression.

To elucidate downstream pathways associated with ALDH2-mediated oxaliplatin sensitivity, transcriptomic profiling was performed using TCGA colorectal cancer datasets stratified by ALDH2 expression. Differential expression analysis followed by gene set enrichment analysis identified multiple negatively enriched pathways in ALDH2-low tumors, including epithelial-mesenchymal transition, inflamematory response, and interferon-related signaling **(Figure 5A-D)**.

Intersection analysis of genes participating in multiple enriched pathways yielded a focused candidate list, which was further screened for associations with oxaliplatin response using ROC plotter analysis. Among the candidates, PDE4B emerged as the only gene whose elevated expression was significantly associated with both oxaliplatin resistance and reduced recurrence-free survival specifically in patients with p53-mutant colorectal cancer **(Figure 5E & 5F)**. In contrast, PDE4B expression showed no prognostic impact in p53-wild-type tumors, suggesting a context-dependent role for PDE4B downstream of ALDH2.

### ALDH2 negatively regulates PDE4B expression in a p53 status-dependent manner

**Figure 6:**
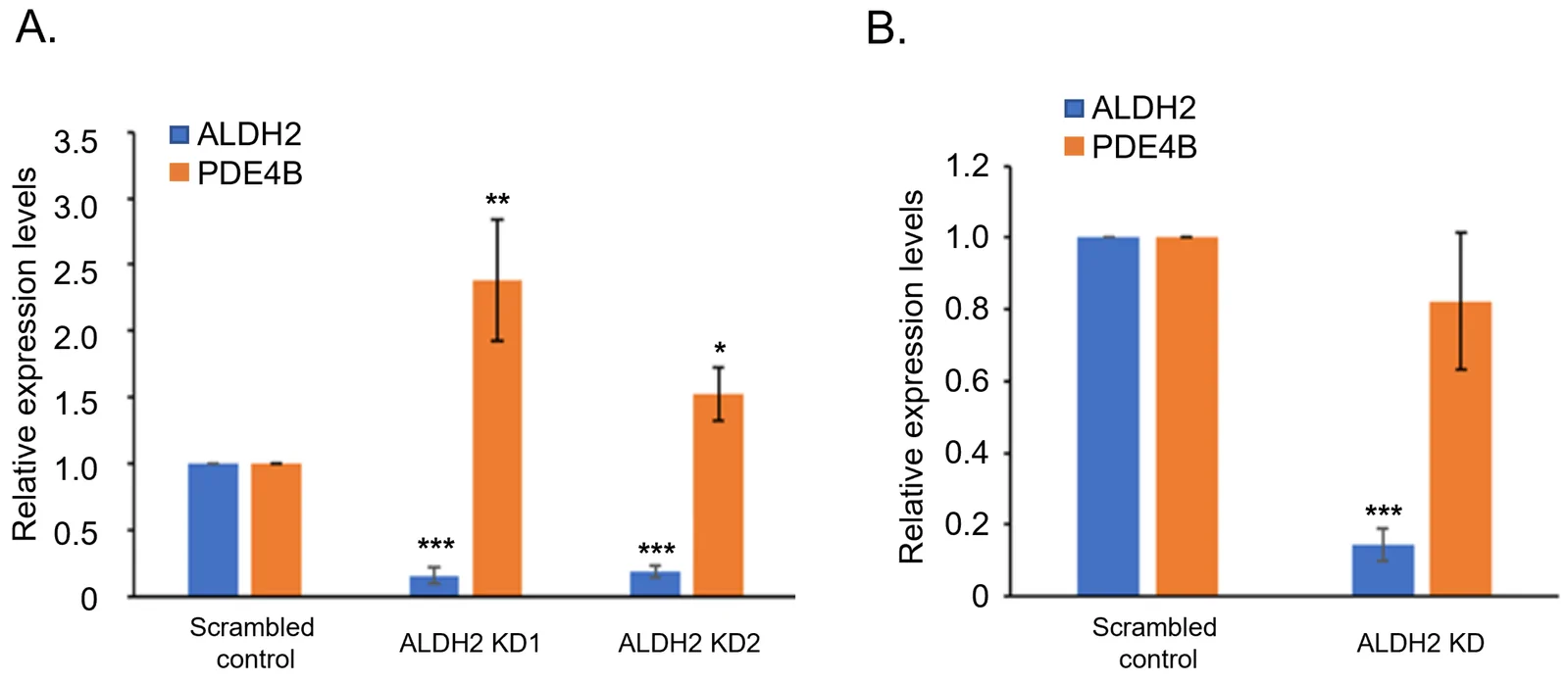
** ALDH2 negatively regulated PDE4B expression in a p53-dependent manner.** (A) DLD-1 cell data indicated that PDE4B underwent notable upregulation following ALDH2 depletion compared to the scrambled control. (B) HCT 116 cells maintained comparable PDE4B expression levels in both ALDH2-knockdown and control conditions. All experiments were conducted in triplicate. *p < 0.05, **p<0.01, ***p<0.001.

Quantitative PCR analysis demonstrated that ALDH2 knockdown resulted in significant upregulation of PDE4B expression in p53-mutant DLD-1 cells **(Figure 6A)**. In contrast, PDE4B expression remained unchanged following ALDH2 depletion in p53-wild-type HCT 116 cells, despite efficient ALDH2 silencing **(Figure 6B)**. These findings indicate that ALDH2-dependent regulation of PDE4B expression is contingent upon p53 status.

### Pharmacological inhibition of PDE4B restores oxaliplatin sensitivity in ALDH2-deficient, p53-mutant CRC cells

**Figure 7:**
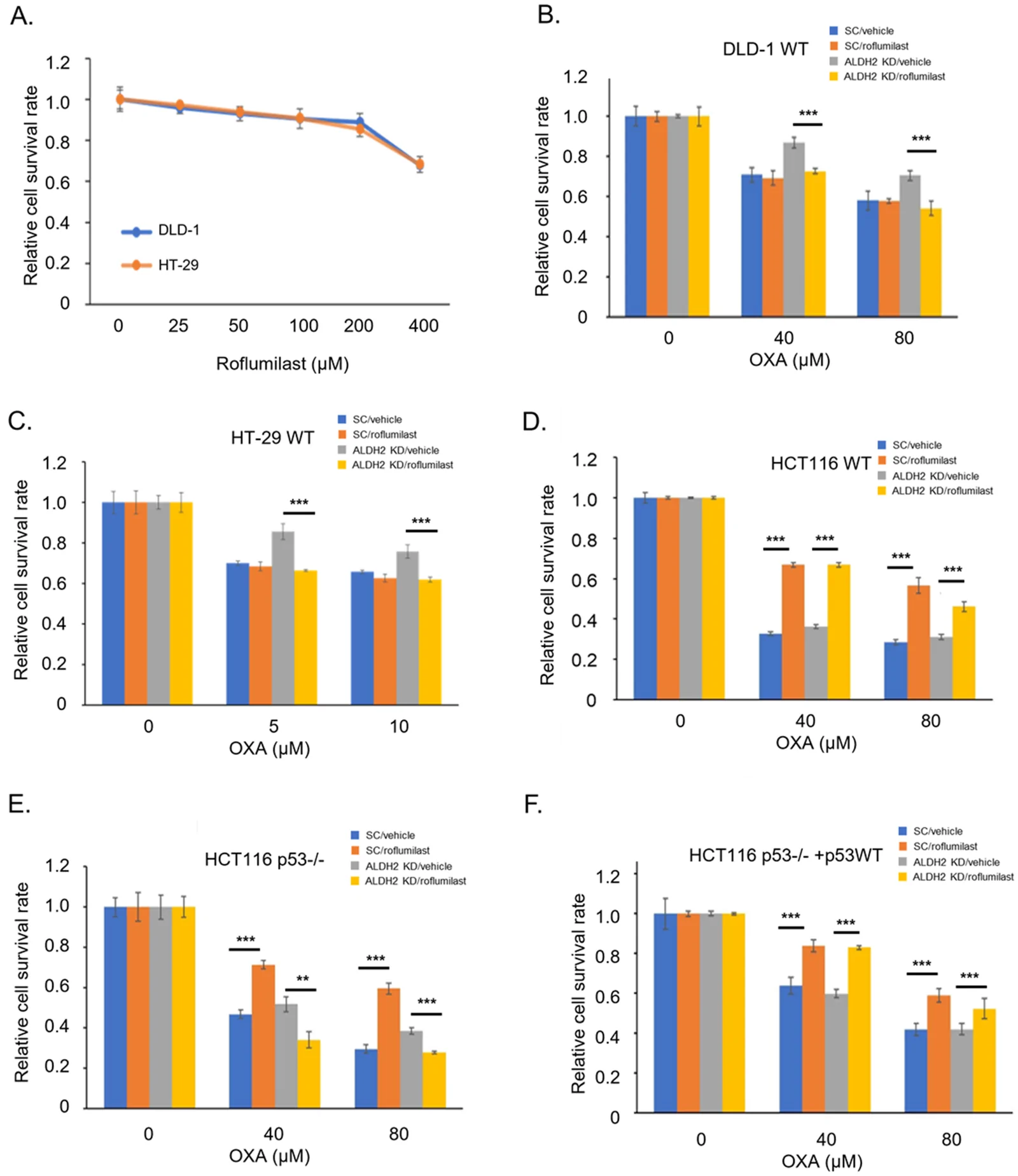
** Inhibition of PDE4B reversed oxaliplatin resistance induced by ALDH2 knockdown in p53 mutation CRC cells.** (A) Cytotoxicity of roflumilast alone was verified. (B & C) Roflumilast substantially reversed the oxaliplatin resistance induced by ALDH2 suppression in DLD-1 and HT-29 cells. (D) In HCT 116 cells, ALDH2 silencing failed to alter oxaliplatin sensitivity, yet roflumilast administration paradoxically enhanced oxaliplatin resistance. (E) Upon ablation of the p53 gene in HCT 116 cells, roflumilast co-treatment with ALDH2 knockdown successfully counteracted the oxaliplatin resistance stemming from ALDH2 depletion. (F) When p53 was reintroduced into HCT 116 p53-/- cells, their pharmacological responses mirrored those of parental HCT 116 cells. All experiments were conducted in triplicate. **p<0.01, ***p<0.001.

To determine whether PDE4B functionally mediates ALDH2-associated oxaliplatin resistance, cells were treated with the PDE4 inhibitor roflumilast in combination with oxaliplatin. Roflumilast alone had minimal effects on cell viability **(Figure 7A)**. However, in ALDH2-depleted DLD-1 and HT-29 cells, roflumilast effectively reversed oxaliplatin resistance and restored drug sensitivity to levels comparable with control cells **(Figure 7B & 7C)**.

In p53-wild-type HCT 116 cells, ALDH2 knockdown did not alter oxaliplatin sensitivity, whereas roflumilast treatment unexpectedly increased resistance **(Figure 7D)**. Importantly, genetic ablation of p53 in HCT 116 cells converted this response pattern, such that roflumilast co-treatment abolished the oxaliplatin resistance induced by ALDH2 depletion **(Figure 7E)**. Re-expression of wild-type p53 reinstated the original response profile **(Figure 7F)**. Together, these data underscore that the functional consequences of PDE4B inhibition are strongly dependent on p53 status.

### PDE4B inhibition rescues oxaliplatin-induced apoptosis in p53-deficient CRC cells

**Figure 8:**
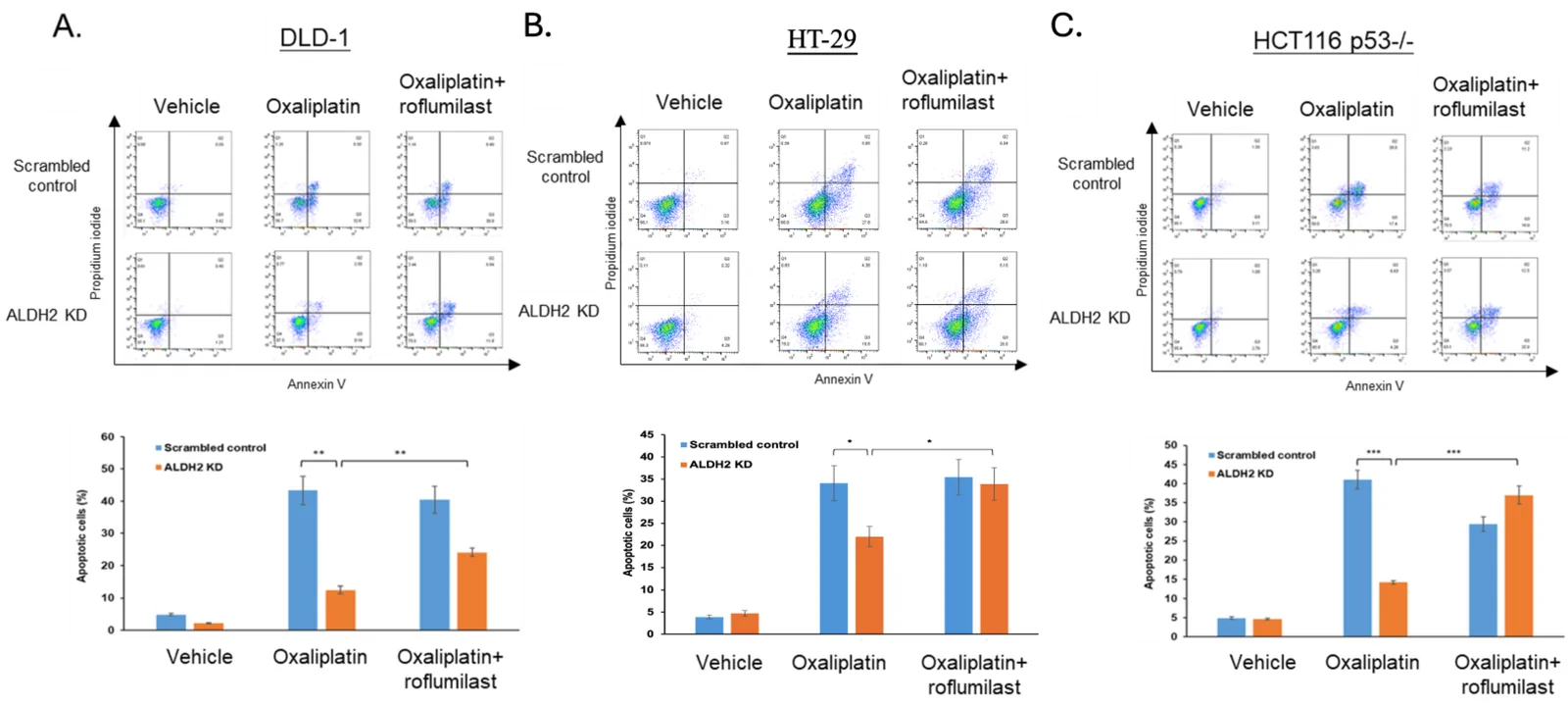
** PDE4B antagonist reversed decreased oxaliplatin-mediated apoptosis induced by ALDH2 knockdown in p53 mutation CRC cells.** (A&B) ALDH2-depleted DLD-1 and HT-29 cells showed less oxaliplatin-induced apoptosis than control. Adding roflumilast enhanced apoptosis beyond oxaliplatin monotherapy. (C) In HCT 116 cells with inactive p53, roflumilast counteracted the protective effect of ALDH2 depletion against oxaliplatin-triggered apoptosis. All experiments were conducted in triplicate. **p<0.01, ***p<0.001.

Finally, apoptotic responses were assessed to confirm the mechanistic basis of restored chemosensitivity. In p53-mutant and p53-deficient CRC cells, ALDH2 knockdown significantly reduced oxaliplatin (80 μM)-induced apoptosis **(Figure 8)**. Co-treatment with roflumilast (100 μM) reinstated apoptotic cell death, as evidenced by increased Annexin V-positive populations **(Figure 8)**. These results demonstrate that PDE4B inhibition restores oxaliplatin sensitivity by reactivating apoptotic signaling in ALDH2-deficient, p53-mutant colorectal cancer cells.

## Discussion

Chemoresistance to platinum-based chemotherapy remains a major clinical challenge in colorectal cancer, particularly in tumors harboring TP53 mutations 17, 18. While p53 dysfunction has long been recognized as a determinant of impaired apoptotic responses, the upstream metabolic regulators and downstream actionable effectors that shape chemotherapy sensitivity in distinct p53 contexts have remained insufficiently defined 18. In this study, we identify a previously unrecognized, p53-dependent ALDH2-PDE4B regulatory axis that governs oxaliplatin sensitivity in colorectal cancer.

Our integrative analyses demonstrate that ALDH2 expression is markedly reduced in colorectal cancer tissues and is associated with adverse clinical outcomes. Importantly, ALDH2 downregulation selectively predicts diminished responsiveness to oxaliplatin, but not to 5-fluorouracil, suggesting a drug-specific relationship rather than a general marker of chemoresistance. These findings extend prior observations implicating ALDH2 in colorectal cancer biology and position ALDH2 as a potential predictive biomarker for platinum-based chemotherapy in molecularly defined patient subsets.

A central finding of this study is that the functional impact of ALDH2 loss on oxaliplatin sensitivity is strictly dependent on p53 status. Both pharmacological inhibition and genetic silencing of ALDH2 consistently induced oxaliplatin resistance in p53-mutant colorectal cancer cells, whereas p53-wild-type cells remained largely unaffected. Genetic ablation and reconstitution experiments further established that loss of functional p53 is sufficient to unmask ALDH2-dependent chemoresistance, while restoration of wild-type p53 reverses this phenotype. These results highlight p53 as a critical contextual determinant that dictates whether ALDH2-mediated metabolic regulation is coupled to chemotherapy-induced apoptosis.

Through transcriptomic profiling and pathway-level analyses, we identified phosphodiesterase 4B (PDE4B) as a key downstream effector associated with ALDH2 loss. Elevated PDE4B expression correlated with oxaliplatin resistance and poor recurrence-free survival specifically in patients with p53-mutant colorectal cancer, underscoring its clinical relevance within this molecular context. Functional experiments further demonstrated that ALDH2 negatively regulates PDE4B expression in a p53-dependent manner, linking metabolic enzyme activity to cAMP-associated signaling pathways that influence cell survival 19.

An important conceptual insight from this work is that the biological consequences of PDE4B modulation are highly context dependent. Pharmacological inhibition of PDE4B using roflumilast effectively restored oxaliplatin sensitivity and apoptotic responses in ALDH2-deficient, p53-mutant colorectal cancer cells. In contrast, PDE4B inhibition exerted distinct, and in some cases opposite, effects in p53-wild-type cells. These findings suggest that p53 status fundamentally shapes the wiring of cAMP-dependent survival signaling, thereby determining whether PDE4B functions as a pro-survival or contextually protective factor.

Although the precise molecular mechanisms by which ALDH2 suppresses PDE4B expression remain to be elucidated, several non-mutually exclusive possibilities may be considered. ALDH2-mediated detoxification of reactive aldehydes may influence redox-sensitive transcriptional programs, while p53 mutations may further reprogram transcriptional or post-transcriptional control of PDE4B. Elucidating the direct molecular intermediates linking ALDH2 activity, p53 status, and PDE4B regulation will be an important direction for future investigation.

ALDH2 deficiency impairs the detoxification of reactive aldehydes such as acetaldehyde and 4-hydroxynonenal, leading to mitochondrial oxidative stress and elevated reactive oxygen species (ROS) production 20. Chronic exposure to acetaldehyde and its associated ROS has been shown to increase PDE4B expression and activity in monocytes, macrophages, and hepatocytes, resulting in markedly reduced intracellular cAMP levels and subsequent attenuation of PKA-dependent anti-inflammatory pathways 21. Furthermore, some adenylyl cyclase isoforms are known to be inhibited by oxidants through cysteine thiol oxidation at their catalytic domains 22, 23. Based on these previous studies, ALDH2 deficiency may impair the detoxification of reactive aldehydes, potentially resulting in oxidative stress and altered cAMP signaling. Although ROS accumulation and intracellular cAMP levels were not directly measured in the present study, these mechanisms provide a biologically plausible framework linking ALDH2 loss to PDE4B upregulation.

To further contextualize these mechanistic findings, previous studies have reported heterogeneous and context-dependent roles of aldehyde dehydrogenase (ALDH) isoforms and phosphodiesterase 4B (PDE4B) in colorectal cancer biology. Distinct ALDH family members have been shown to exert divergent effects on chemotherapy response depending on cellular context and therapeutic stress 24, 25. Durinikova *et al.* reported that 5-FU-resistant HT-29 cells exhibited decreased ALDH2 expression concomitant with increased ALDH1A3 expression, suggesting that distinct ALDH isoforms may exert divergent, and even opposing, effects on chemoresistance 26. PDE4B has also been implicated in colorectal tumorigenesis in both pro-tumorigenic and contextually protective roles across disease stages 27, 28. Our observations that PDE4B modulation exerts opposing effects on oxaliplatin sensitivity depending on p53 status provide a unifying framework that helps reconcile these seemingly divergent reports and highlight the importance of molecular context in interpreting PDE4B function.

Phosphodiesterase 4B (PDE4B) has been implicated in colorectal cancer (CRC) progression through oncogenic signaling pathways. Kim *et al.* demonstrated that PDE4B is highly expressed in CRC tissues and that pharmacological inhibition of PDE4B using roflumilast suppresses tumor growth via the PDE4B-mTOR-MYC axis 28. Earlier work by Tsunoda *et al.* further showed that oncogenic KRAS upregulates PDE4B expression and that PDE4B inhibition induces luminal apoptosis in CRC models 29. In contrast, other studies reported that PDE4B may play a protective role in colorectal adenoma or early cancer and becomes epigenetically silenced during CRC progression 30, 31, suggesting that PDE4B function may be stage-dependent. Notably, however, the involvement of PDE4B in chemotherapy resistance has not been previously explored.

Mutations in p53 have been widely reported to contribute to resistance to multiple chemotherapeutic agents, including fluoropyrimidines and platinum-based drugs 8. Mechanistically, p53 mutations can impair chemotherapy-induced apoptosis through inactivation of PUMA transcription 32, 33 or promote drug resistance via downstream effectors such as LRPPRC, as demonstrated in the miR-34a/LRPPRC/MDR1 axis mediating 5-FU resistance 34. Consistent with these findings, our results further extend the role of p53 by showing that p53 status not only influences canonical DNA damage responses but also dictates the downstream effects of specific metabolic-signaling interactions.

Collectively, this study identifies a p53 status-dependent ALDH2-PDE4B regulatory axis that modulates chemotherapy sensitivity in CRC. These findings provide new insight into how p53 mutations may reprogram therapeutic responses through selective metabolic enzyme-signaling crosstalk and highlight the context-dependent roles of ALDH2 and PDE4B in colorectal cancer treatment.

This study has several limitations. First, the findings are primarily based on *in vitro* models and analyses of public clinical datasets, and therefore do not fully capture the complexity of the tumor microenvironment. The translational significance of the ALDH2-PDE4B axis has not yet been validated in xenograft or patient-derived xenograft models, while such *in vivo* studies will be essential to confirm the clinical relevance. Second, although p53 dependency was rigorously validated using genetic approaches, it remains unclear whether the ALDH2-PDE4B axis operates similarly across distinct p53 mutation subtypes, as different gain-of-function or loss-of-function p53 mutations may confer divergent functional consequences. Additional p53-mutant colorectal cancer models may further refine the generalizability of these observations. Third, the proposed pathway whereby ALDH2 deficiency promotes oxidative stress, leading to PDE4B upregulation and subsequent cAMP suppression, remains inferential and requires direct experimental validation in CRC models. Fourth, the high drug concentrations employed in the experiments may limit direct clinical extrapolation of the pharmacological findings. Additionally, daidzein as a pharmacological ALDH2 inhibitor may exert off-target effects including estrogen receptor activation and tyrosine kinase inhibition. Thus, its results should be interpreted in conjunction with the genetic silencing data presented in this study. PDE4B protein levels were not directly assessed in this study; future protein-level quantification will be important to corroborate the transcriptional findings and to more fully characterize the regulatory relationship between ALDH2 and PDE4B. Finally, prospective clinical validation will be required to establish the utility of ALDH2 and PDE4B as predictive biomarkers in patient stratification.

Despite these limitations, our findings have important translational implications. Roflumilast is an FDA-approved PDE4 inhibitor with an established safety profile 35, 36, raising the possibility of therapeutic repurposing to overcome oxaliplatin resistance in molecularly selected colorectal cancer patients. Our data suggest that patients with p53-mutant tumors exhibiting low ALDH2 expression may represent a subgroup most likely to benefit from such combination strategies. Further preclinical and clinical studies are warranted to evaluate this therapeutic concept.

In conclusion, this study defines a novel p53 status-dependent ALDH2-PDE4B axis that regulates oxaliplatin sensitivity in colorectal cancer. By integrating clinical data analyses with functional genetic and pharmacological approaches, we provide mechanistic insight into how metabolic regulation and cAMP signaling intersect with p53 status to shape chemotherapy response. These findings advance our understanding of context-dependent chemoresistance mechanisms and provide a rationale for evaluating PDE4B inhibition as a therapeutic strategy in p53-mutant colorectal cancer.

## Supplementary Material

Supplementary figures and tables.

## Data Availability

The dataset supporting the conclusions of this article is included within the article.
