# Supplementary material for: ALDH2 regulates oxaliplatin sensitivity via PDE4B in p53-mutant colorectal cancer
Source: Int J Med Sci. 2026 Jul 27;23(9):2835–46. doi: 10.7150/ijms.133461 (PMC13539918; doi:10.7150/ijms.133461)
Supplement: Supplementary file 1 [file ijmsv23p2835s1.pdf]

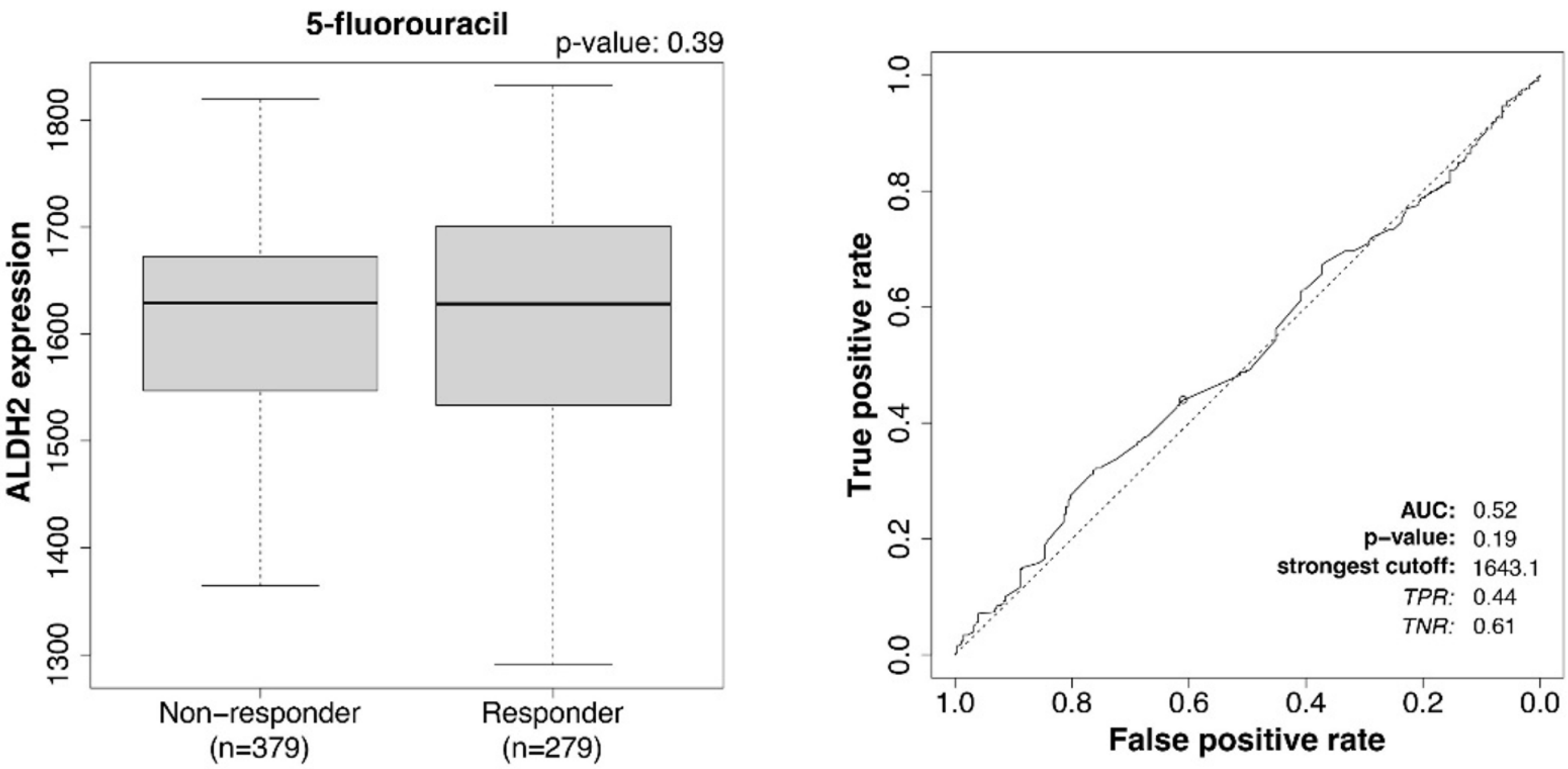

Figure S1. ALDH2 expression is not correlated with 5-fluorouracil response in CRC.
